# CircHIPK3 Promotes Pyroptosis in Acinar Cells Through Regulation of the miR-193a-5p/GSDMD Axis

**DOI:** 10.3389/fmed.2020.00088

**Published:** 2020-04-07

**Authors:** Jiale Wang, Xia Li, Yunfei Liu, Cheng Peng, Hongwei Zhu, Guangping Tu, Xiao Yu, Zhiqiang Li

**Affiliations:** Department of Hepatobiliary and Pancreatic Surgery II, The Third Xiangya Hospital, Central South University, Changsha, China

**Keywords:** acute pancreatitis, circular RNA, pyroptosis, microRNA, gasdermin family

## Abstract

Acute pancreatitis (AP), especially severe acute pancreatitis (SAP), is an extremely dangerous illness with a high mortality rate. Pyroptotic cells release their cellular contents and inflammatory factors, aggravating the inflammatory response. Pyroptosis may be the main mode of acinar cell death during AP. The circular RNA circHIPK3 is expressed in pancreatic tissue and is associated with inflammatory response. In this study, we focused on the role and underlying mechanism of circHIPK3 in AP. We found that the expression of circHIPK3 was significantly elevated in serum of patients with AP and in caerulein-stimulated AR42J cells and was associated with caspase-1 and caspase-11 activation. circHIPK3 silencing ameliorated caerulein-induced cell damage and reduced the release of inflammatory factors IL-1β, IL-6, IL-8, and TNF-α and inhibited the activation of caspase-1 and caspase-11. In addition, circHIPK3 bound to miR-193a-5p and negatively regulated its expression. Inhibition of miR-193a-5p increased the release of IL-1β, IL-6, IL-8, and TNF-α and activated caspase-1 and caspase-11, thereby counteracting the effect of circHIPK3 silencing on caerulein-induced cell damage. Furthermore, we identified GSDMD as a target gene of miR-193a-5p, which is the key gene for pyroptosis. Interfering with the expression of GSDMD can increase cell viability, reduce the secretion of inflammatory cytokines, and suppress the activation of cleaved caspase-1 and caspase-11. Silencing GSDMD reversed the effects of miR-193a-5p inhibitors on caerulein-induced damage. In conclusion, circHIPK3 promotes pyroptosis in acinar cells through regulation of the miR-193a-5p/GSDMD axis, which eventually aggravates AP disease.

## Introduction

The occurrence and development of acute pancreatitis (AP) have been the focus of research in the field of surgery (1). The condition of AP, especially severe acute pancreatitis (SAP), is dangerous and has a high mortality rate (2). In the early stage of AP, activated pancreatic enzymes, excess oxygen free radicals, and pro-inflammatory cytokines cause damage and death of acinar cells (3). The types of death in acinar cells during SAP include apoptosis, necrosis, and pyroptosis (4). When acinar cells are mainly apoptotic, the clinical manifestations are mild edematous pancreatitis, which has good prognosis (5). When damaged by pyroptosis, acinar cells are ruptured and release the cellular contents and inflammatory factors, such as tumor necrosis factor alpha (TNF-α) and IL-1β, aggravating the inflammatory response (6). Therefore, this mode of acinar cell death has always been a hot spot in AP research.

A previous study showed that the morphology of acinar cells during AP had the characteristics of pyroptosis (7). Pyroptosis is a caspase-1/4/5/11 mediated programmed cell death (8). Caspase-1 activation is also present also in AP acinar cells, suggesting that pyroptosis is associated with AP (7). Understanding the mechanisms of pyroptosis in acinar cells will help to provide new ideas for clinical diagnosis and treatment of AP.

Circular RNAs (circRNAs) are a class of RNAs with a unique closed-loop structure that are stably expressed in many biological cells (9). Studies have found that circRNAs can regulate gene expression levels at various levels by binding to miRNAs and proteins and regulating protein translation and modification and are involved in many biological processes, such as cell differentiation, proliferation, and apoptosis (10, 11). Therefore, circRNAs play an important regulatory role in the development of diseases, such as cardiovascular diseases, tumors, and autoimmune diseases (12). For example, circRNA caspase-1-associated circRNA (CACR) is upregulated in high-glucose-treated cardiomyocytes. CACR silencing inhibits high-glucose-induced caspase-1 activation and pyroptosis in cardiomyocytes (11), suggesting that circRNAs are associated with pyroptosis.

In this study, we focused on the role and underlying mechanism of circHIPK3 in AP. circHIPK3 (hsa_circ 0000284) is a circular RNA derived from the second exon of the HIPK3 gene. circHIPK3 is highly expressed in liver, brain, and lung (13–15) and has shown an important role in the regulation of cell survival, oxidative damage, and inflammation (16–18). We note that circHIPK3 is also expressed in pancreatic tissue (19). In addition, circHIPK3 silencing has been shown to inhibit the release of IL-1β and TNF-α, indicating a close relationship between circHIPK3 and inflammation in AP (20). However, the role and mechanism of circHIPK3 in AP remains unclear. In this study, our results showed that circHIPK3 was highly expressed in the serum of AP patients and in caerulein-stimulated AR42J cells. In addition, we demonstrated that circHIPK3 silencing attenuated caerulein-mediated pyroptosis in AR42J cells. Furthermore, mechanistic study showed that that circHIPK3 promoted pyroptosis and inflammation through regulation of the miR-193a-5p/GSDMD axis in AR42J cells. Therefore, circHIPK3 could become a new biomarker and therapeutic target in AP.

## Materials and Methods

### Human Sample Collection

During the period from March 2017 to December 2018, a total of 72 patients with acute pancreatitis (50 males and 22 females; age: 30–72 years; mean age: 48.3 years) were included in the study. The diagnostic criterion of AP diagnosis is to meet the two of the following conditions: (1) acute, persistent, severe and unbearable upper abdominal pain, often radiated to the back; (2) serum amylase and/or lipase activity is three times or more higher than the normal upper limit; (3) enhanced CT/MRI or abdominal ultrasound showed AP imaging changes.

The diagnostic criteria of MAP diagnosis are to meet the above AP diagnosis and one of the following conditions: (1) no organ dysfunction, no complications, Ranson score <3 points; (2) acute physiology and chronic health evaluation (APACHE II) score <8 points; (3) bedside index of severity in acute pancreatitis (BISAP) score <3 points; (4) improved CT is severe. The modified CT severity index (MCTSI) score was <4 points.

The diagnostic criteria of SAP diagnosis are to meet the above AP diagnosis and one of the following conditions: (1) Ranson score ≥3 points; (2) APACHE II score ≥8 points; (3) BISAP score ≥3 points; (4) MCTSI score ≥4 points; (5) organ dysfunction (duration <48 h); (6) complications requires intervention during the recovery period.

Volunteers who passed the physical examination at the physical examination center of our hospital were included in this study. The criteria are as follows: blood routine, lipase, amylase, myocardial enzyme, erythrocyte sedimentation rate, C-reactive protein, electrocardiogram, chest X-ray, blood sugar, infectious diseases, thyroid function, and abdominal color Doppler ultrasound were not abnormal and no abdominal pain symptoms.

Exclusion Criteria: patients with chronic diseases, such as diabetes, cardiovascular disease, chronic pancreatitis, hepatitis, and malignant tumors would be excluded from the study. Blood samples were obtained from SAP patients, MAP patients, and healthy people. The study was approved by the Clinical Research Ethics Committee of the Third Xiangya Hospital, Central South University, and all participants signed informed consent forms.

### Cell Culture

Rat pancreatic acinar AR42J cells were purchased from the Chinese Academy of Sciences cell bank (Beijing, China). These cells were cultured in F-2K medium supplemented with 50 μg/ml streptomycin, 50 IU penicillin, and 20% FBS at the conditions of 5% CO_2_ and 95% O_2_ at 37°C.

To establish an acute pancreatitis model *in vitro*, AR42J cells were treated with 10 nM caerulein (Sigma-Aldrich, St. Louis, MO) for different time periods, specifically 0, 4, 6, 8, and 10 h.

### Cell Transfection

For shRNA-mediated circHIPK3 and GSDMD gene knockdown, 1 × 10^6^ AR42J cells were seeded in 6-well plates to 80% confluency and then transfected. In order to selectively target to circHIPK3, the shRNA sequences of circHIPK3 were designed at the head-to-tail junction (backsplice junction) to avoid complementary pairing to the linear mRNA (7-mer seed not present in the linear host gene). The control sequence was matched on one end of the backsplice junction and was mismatched on the other end. The shRNA sequence used in this study was synthesized by GenePharma (Shanghai, China) and packaged into hU6-MCS-PGK-EGFP lentiviral vector (GenePharma, Shanghai, China). The multiplicity of infection is 100. Transfection efficiency was determined by FACScalibur flow cytometry (Becton Dickinson, Franklin Lakes, NJ, USA) with a transfection efficiency of over 90%. For miRNA inhibitor and mimic transfection, miR-193a-5p inhibitor and mimic were purchased from Guangzhou Ribo Biotech (Guangzhou, China) and transfected according to the manufacturer's instructions.

### Quantitative Polymerase Chain Reaction

Total RNA was extracted from cells and then reverse transcripted to produce complementary DNA (cDNA) using a high-capacity cDNA reverse transcription kit (Applied Biosystems, California). The cDNA of the miRNA was generated using the TaqMan miRNA Reverse Transcription Kit (Applied Biosystems, Foster City, California, USA). Subsequently, qPCR was used to detect the expression of circHIPK3, HIPK3, and GSDMD by TaqMan gene detection kit, or miR-30a, miR-124, and miR-193a-5p expression were detected using TaqMan Universal Master Mix II. Since circular RNA is resistant to RNase R, we used RNase R treatment to reduce the effect of linear PCR products on circular RNA. GAPDH and U6 were used as internal controls for RNA and miRNA, respectively. The following primers were used: circHIPK3, forward 5′-TGGAGACTGGGGGAAGATGA-3′ and reverse 5′-CACACTAACTGGCTGAGGGG-3′; HIPK3, forward 5′-GACCTGAGGAGATCAAGCCG-3′ and reverse 5′-ACTCCTCACTCTTGCGCTTC-3′; GSDMD, forward 5′-CTCGCCGACTTCCGTAAACT-3′ and reverse 5′-TCCAGCGATCCTGGGTTCTA-3′; GAPDH, forward 5′-CATGAGAAGTATGACAACAGCCT-3′ and reverse 5′-AGTCCTTCCACGATACCAAAGT-3′.

### Enzyme-Linked Immunoassay (ELISA)

The concentrations of IL-1β, IL-6, IL-8, and TNF-α were determined by a commercial enzyme-linked immunoassay kit (ELISA, Sangon Biotech) according to the procedure provided by the manufacturer. Results are expressed in picograms per milliliter. Amylase enzyme activity was also determined by a commercially available enzyme-linked immunoassay kit (cat no. K711-100, BioVision) according to the procedure provided by the manufacturer.

### Propidium Iodide Staining

Propidium iodide (PI) cannot penetrate intact cell membranes, so normal cells or apoptotic cells with intact cell membranes cannot be stained. However, PI can stain the pyroptotic cells that lost the integrity of cell membranes. Briefly, AR42J cells were cultured on coverslips to adherence and 80% confluence. The cells were washed in PBS and fixed in pre-cooled 70% ethanol for 30 min at 4°C and then treated with 50 μL ribonuclease (100 μg/mL) for 20 min. Finally, 200 μL of PI (final concentration 50 μg/mL, Beyotime Biotechnology, Shanghai, China) was added to incubate for 30 min in the dark, and then PI staining was observed with a fluorescence microscope, and the PI-positive cells were photographed and counted.

### Flow Cytometry

The AR42J cells were treated with caerulein for 8 h. The untreated cells were used as control. After intensive washing with PBS containing 2% (wt/vol) FBS, cells were stained with fluorochrome-conjugated antibodies [anti–caspase-1 (AG-20B-0042-C100) was from Adipogen; anti–caspase-11 (NB120-10454) was from Novus] for FACS analysis. All flow cytometry was performed on an FACScalibur flow cytometer (Becton Dickinson, Franklin Lakes, NJ, USA), and data were analyzed by FlowJo 7.6.1 software.

### Dual-Luciferase Reporter Gene Assay

The wild type 3′UTR and mutant 3′UTR of GSDMD were synthesized by Sangon Biotech using the Site-Directed Mutagenesis Kit (Sangon Biotech (Shanghai) Co., Ltd., Shanghai, China) and inserted into the pGL3-LUC report vector (Promega). Samples of 2 × 10^5^ AR42J cells were seeded in 24-well plates for 12 h, and 100 ng of blank vector or GSDMD WT 3′UTR and MUT 3′ UTR vectors and 400 ng of LUC reporter vector were co-transfected into the cells for 48 h. After 48 h of transfection, cells were harvested and assayed for luciferase activity using Dual-Luciferase Assay (Promega) according to the manufacturer's instructions. Co-transfection with the pRL-TK Renilla Luciferase Reporter Vector (Promega) was used as a control.

### Western Blot Analysis

The treated cells were lysed in RIPA buffer containing 1 mM DTT, 11 μg/ml DNase I, and protease inhibitor cocktail (Roche) and incubated on ice for 30 min. Sixty micrograms of protein sample were separated by electrophoresis on a denatured 10% SDS-PAGE gel and blotted onto a PVDF membrane (Millipore). The primary antibodies (Anti-Caspase-1, 1:1,000, ab138483, Abcam; Anti-cleavage Caspase-1, 1:1,000, ab207802, Abcam; Anti-Caspase-11, 1:1,000, ab22684, Abcam Anti-cleavage Caspase-11, 1:1,000, ab180673, Abcam) were incubated overnight at 4°C. Rabbit monoclonal to GAPDH (EPR16891, Abcam) was used as a loading control. Afterward, the membranes were rinsed and subsequently incubated with appropriate secondary antibodies. An ECL kit (Beyotime Biotechnology, Shanghai, China) was used to detect the bands of Western blots.

### CCK-8 Analysis of Cell Viability

For cell viability assays, the transfected cells were seeded into 96-well plates (3,000 cells/well), and cell viability was assessed using a CCK-8 kit (Beyotime Biotechnology, Shanghai, China) according to the manufacturer's instructions. Briefly, after transfection, the cells were seeded into 96-well plates (3,000 cells/well) and cultured for 24 h. Then, 10 μl of CCK-8 solution was added to the cell culture medium and incubated for another 4 h. Absorbance at a wavelength of 450 nm was detected in a microplate reader (ELx808, BioTek, USA).

### Statistical Analysis

Each experiment in this study was repeated three times independently. Data are expressed as mean ± standard deviation (SD). SPSS Statistics 20.0 software (IBM, Armonk, NY, USA) was used for data analysis. Analysis of variance or two-tailed Student's *t*-test was used to calculate differences between groups. *P* < 0.05 was considered statistically significant.

## Result

### CircHIPK3 Is Highly Expressed in Serum Samples of Patients With Acute Pancreatitis

**Figure 1:**
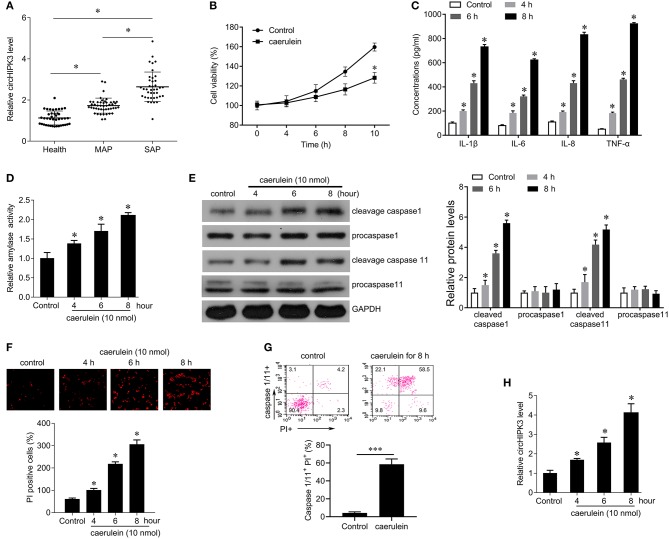
The expression of circHIPK3 in serum samples of patients with AP and in caerulein-stimulated pancreatic acinar cells. **(A)** QPCR was performed to detect circHIPK3 expression in serum samples of patients with AP and healthy subjects. MAP, mild acute pancreatitis; SAP, severe *Acute Pancreatitis*. **(B)** CCK8 assay was performed to measure the cell viability in AR42J cells after caerulein treatment. **(C)** Levels of inflammatory cytokines IL-1β, IL-6, IL-8, and TNF-α were measured by ELISA kits in culture medium after caerulein treatment. **(D)** Amylase activity was measured by ELISA kit in AR42J cells after caerulein treatment. **(E)** The pyroptosis-related proteins caspase 1 and caspase 11 were analyzed by immunoblot in AR42J cells after caerulein treatment. **(F)** PI staining was performed in AR42J cells after caerulein treatment, and the PI-positive cells were counted. **(G)** Flow-cytometric analysis of caspase-1/11^+^PI^+^ cells in AR42J cells after caerulein treatment. Data are presented as a representative plot (upper) and quantified percentages (lower). **(H)** circHIPK3 expression was determined by qPCR in AR42J cells after caerulein treatment. *p < 0.05.

Of the 72 patients with AP included in this study, 61 had pancreatic enlargement, including 49 with diffuse pancreatic swelling, 6 with pancreatic head enlargement, and 6 with pancreatic body and tail enlargement, while 11 had normal pancreas size. According to the clinical severity score, there were 38 SAP patients and 34 MAP patients in the 72 patients with AP. In addition, 34 healthy volunteers were recruited as normal controls. Compared with the healthy control group, the expression level of circHIPK3 was significantly increased in AP, and the level of circHIPK3 in SAP patients was significantly higher than that in MAP patients (Figure 1A), suggesting that the expression of circHIPK3 is associated with the severity of the disease.

To investigate the role of circHIPK3 in acute pancreatitis, we constructed a model of acute pancreatitis *in vitro* by using caerulein to stimulate AR42J cells for different time periods. The results showed that caerulein significantly reduced cell viability (Figure 1B), enhanced the secretion of the inflammatory cytokines IL-1β, IL-6, IL-8, and TNF-α (Figure 1C), and increased the activity of amylase in a time-dependent manner (Figure 1D) compared with controls. In addition, we found that caerulein stimulation resulted in a significant increase in the number of PI-positive cells, suggesting that the membrane integrity of AR42J cells was disrupted (Figure 1F). We further examined the expression of caspase-1 and caspase-11 and found that caerulein treatment significantly increased the expression of cleavage capase1 and cleavage caspase-11, suggesting that caerulein treatment may induce AR42J cell pyroptosis (Figure 1E). FACS revealed a marked increase of caspase-1/11^+^ propidium iodide (PI)^+^ cells gated on the AR42J cells treated with caerulein for 8 h compared with control [(58.5 vs. 4.2%), Figure 1G]. Furthermore, we examined the expression level of circHIPK3 and observed that caerulein treatment significantly increased the expression of circHIPK3 in a time-dependent manner (Figure 1H). Since the damage to the AR42J cells induced by caerulein was most obvious at the 8-h time point, that time point was selected for subsequent experiments. Collectively, these data suggest that circHIPK3 and pyroptosis are associated with acute pancreatitis.

### Silencing circHIPK3 Expression Attenuates Caerulein-Induced Damage in AR42J Cells

**Figure 2:**
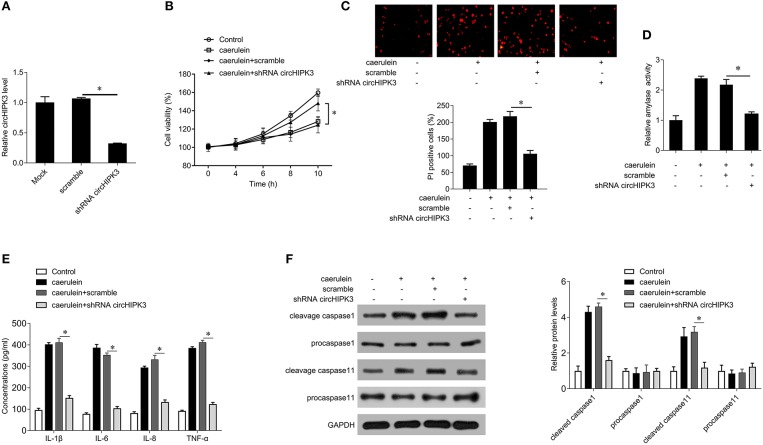
circHIPK3 knockdown inhibits caerulein-mediated cell death in AR42J cells. **(A)** circHIPK3 levels were analyzed by qPCR after shRNA transfection in AR42J cells. **(B)** CCK8 assay was performed to measure the cell viability in AR42J cells after caerulein treatment or with shRNA transfection. **(C)** PI staining was performed to determine the cell pyroptosis after caerulein treatment or with shRNA transfection in AR42J cells. **(D)** Amylase activity was measured by ELISA kit after caerulein treatment or with shRNA transfection in AR42J cells. **(E)** Levels of inflammatory cytokines IL-1β, IL-6, IL-8, and TNF-α were measured by ELISA kits in culture medium after caerulein treatment or with shRNA transfection. **(F)** The pyroptosis-related proteins caspase 1 and caspase 11 were analyzed by immunoblot after caerulein treatment or with shRNA transfection in AR42J cells (left) and the bands were quantified (right). *p < 0.05.

In order to explore the effect of circHIPK3 on AP, we silenced circHIPK3 in AR42J cells with lentivirus packed with interference sequences and then stimulated AR42J cells with caerulein. shRNA transfection significantly decreased the level of circHIPK3 compared with the scramble group (Figure 2A) but did not alter the expression of host gene HIPK3 (Figure S1). Subsequent experiments showed that silencing circHIPK3 increased cell viability (Figure 2B) and reduced the number of PI-positive cells (Figure 2C), suppressed amylase activity (Figure 2D), and inhibited the secretion of the inflammatory cytokines IL-1β, IL-6, IL-8, and TNF-α (Figure 2E). Furthermore, silencing of circHIPK3 significantly reduced the expression of cleaved caspase-1 and caspase-11. These results indicate that silencing circHIPK3 significantly attenuates caerulein-induced damage in AR42J cells and is associated with pyroptosis.

### miR-193a-5p Inhibitors Reverse the Effects of circHIPK3 Silencing on Caerulein-Induced Injury in AR42J Cells

**Figure 3:**
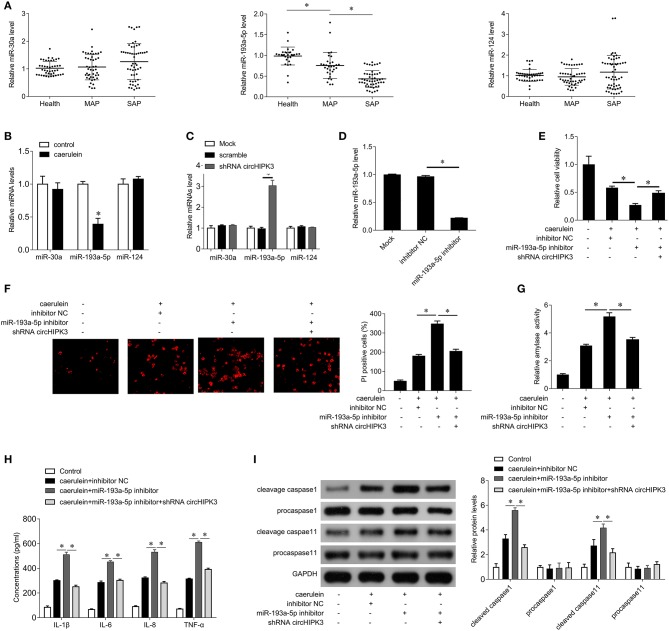
circHIPK3 acts as a miR-193a-5p sponge. **(A)** QPCR was performed to detect the expression of miR-30a, miR-193a-5p, and miR-124 in serum samples of patients with AP and healthy subjects. MAP, mild acute pancreatitis; SAP, severe acute pancreatitis. **(B)** qPCR was performed to determine the expression of miR-30a, miR-193a-5p, and miR-124 in AR42J cells after caerulein treatment. **(C)** qPCR was performed to determine the expression of miR-30a, miR-193a-5p, and miR-124 after shRNA circHIPK3 transfection in AR42J cells. **(D)** qPCR was performed to determine the expression of miR-193a-5p after miR-193a-5p inhibitor transfection in AR42J cells. **(E)** CCK8 assay was performed to measure the cell viability in AR42J cells after caerulein treatment or with shRNA transfection and miR-193a-5p inhibitor transfection. **(F)** PI staining was performed to determine the cell pyroptosis after caerulein treatment or with shRNA transfection and miR-193a-5p inhibitor transfection. **(G)** Amylase activity was measured by ELISA kit after caerulein treatment or with shRNA transfection and miR-193a-5p inhibitor transfection in AR42J cells. **(H)** Levels of inflammatory cytokines IL-1β, IL-6, IL-8, and TNF-α were measured by ELISA kits in culture medium after caerulein treatment or with shRNA transfection and miR-193a-5p inhibitor transfection. **(I)** The pyroptosis-related proteins caspase 1 and caspase 11 were analyzed by immunoblot after caerulein treatment or with shRNA transfection and miR-193a-5p inhibitor transfection in AR42J cells (left), and the bands were quantified (right). *p < 0.05.

Previous studies have shown that circHIPK3 plays a role in the regulation of multiple miRNAs, including miR-30a, miR-124, and miR-193a-5p, which are closely related to inflammation (16). Therefore, we examined the expression of miR-30a, miR-124, and miR-193a-5p in AP patients. As shown in Figure 3A, there was no significant change in the expression of miR-30a and miR-124 compared with the healthy group, while the expression of miR-193a-5p was significantly decreased in AP patients. The level of miR-193a-5p in SAP patients was significantly lower than that of MAP patients. Caerulein treatment also significantly inhibited the expression of miR-193a-5p but had no significant effect on the expression of miR-30a and miR-124 (Figure 3B). Furthermore, silencing of circHIPK3 significantly increased the expression of miR-193a-5p but did not alter the expression of miR-30a and miR-124 (Figure 3C). These results suggest that circHIPK3 may play a role in AP through regulating miR-193a-5p. To further demonstrate this hypothesis, we used the miR-193a-5p inhibitor to inhibit the expression of endogenous miR-193a-5p in AR42J cells (Figure 3D). The results showed that inhibition of miR-193a-5p expression enhanced caerulein-induced inhibition of cell viability (Figure 3E), promoted cell pyroptosis (Figure 3F), enhanced amylase activity (Figure 3G), increased secretion of inflammatory cytokines IL-1β, IL-6, IL-8, and TNF-α (Figure 3H), and increased expression of cleaved caspase 1 and caspase 11 (Figure 3I). Our abovementioned results indicate that silencing circHIPK3 significantly attenuates caerulein-induced damage (Figures 2B–F), while inhibiting miR-193a-5p reverses the effect of silencing circHIPK3 on caerulein-induced injury (Figures 3E–I). These results indicate that silencing of circHIPK3 significantly attenuates caerulein-induced damage in AR42J cells by enhancing miR-193a-5p function and is associated with decreased cell pyroptosis.

### miR-193a-5p Targets GSDMD

**Figure 4:**
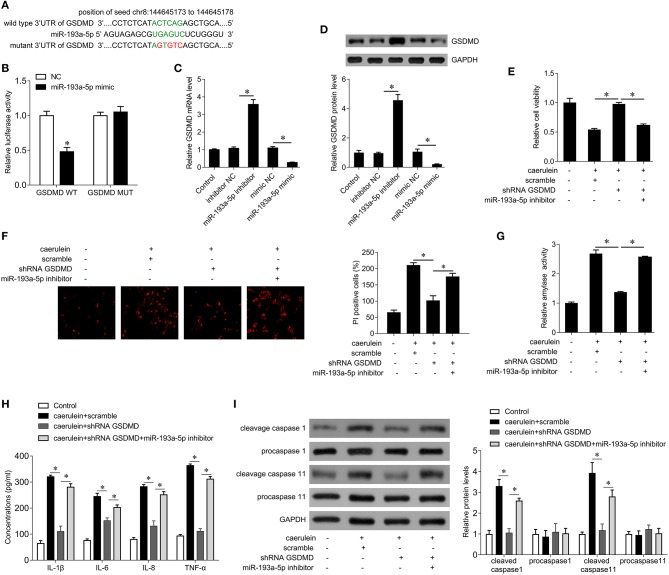
miR-193a-5p inhibits cell pyroptosis by targeting GSDMD in AR42J cells. **(A)** The binding site of miR-193a-5p on the 3′ UTR of GSDMD. The complementary bases are in green, and the mutant sites are red. **(B)** Wild type GSDMD 3′ UTR or mutant 3′ UTR as well as miR-193a-5p mimics were transfected into AR42J cells, and then the dual-luciferase assay was performed. *p < 0.05 vs. Mimic-NC. **(C)** The mRNA expression of GSDMD was analyzed by qPCR in AR42J cells after the indicated treatment. **(D)** The protein expression of GSDMD was analyzed by Western blotting in AR42J cells after the indicated treatment (upper), and the bands were quantified (lower). **(E)** CCK8 assay was performed to measure the cell viability in AR42J cells after caerulein treatment or with miR-193a-5p inhibitor transfection and shRNA GSDMD transfection. **(F)** PI staining was performed to determine the cell pyroptosis after caerulein treatment or with miR-193a-5p inhibitor transfection and shRNA GSDMD transfection in AR42J cells. **(G)** Amylase activity was measured by ELISA kit after caerulein treatment or with miR-193a-5p inhibitor transfection and shRNA GSDMD transfection in AR42J cells. **(H)** Levels of inflammatory cytokines IL-1β, IL-6, IL-8, and TNF-α were measured by ELISA kits in culture medium after caerulein treatment or with miR-193a-5p inhibitor transfection and shRNA GSDMD transfection. **(I)** The pyroptosis-related proteins caspase 1 and caspase 11 were analyzed by immunoblot after caerulein treatment or with miR-193a-5p inhibitor transfection and shRNA GSDMD transfection in AR42J cells (left), and the bands were quantified (right). *p < 0.05.

We further explored the target of miR-193a-5p to explore possible regulatory patterns of miR-193a-5p in AP. GSDMD is predicted to be a potential target for miR-193a-5p, and GSDMD is a key gene that promotes cell pyroptosis. The complementary sequences between GSDMD and miR-193a-5p are shown in Figure 4A. To verify the binding sites between them, we performed a luciferase reporter gene assay. The results showed that the miR-193a-5p mimic significantly inhibited the luciferase activity of GSDMD-wt, while the luciferase activity of GSDMD-mut remained unchanged (Figure 4B), confirming that GSDMD is a target gene of miR-193a-5p. In addition, miR-193a-5p mimics significantly inhibited GSDMD mRNA and protein expression levels, while miR-193a-5p inhibitors significantly increased GSDMD mRNA and protein expression levels (Figures 4C,D), suggesting that miR-193a-5p negatively regulates GSDMD expression.

We next further demonstrated whether miR-193a-5p inhibits caerulein-induced AR42J cell damage by negatively regulating GSDMD. The results showed that interference with GSDMD expression attenuated the inhibition of cell viability by caerulein treatment (Figure 4E), inhibited cell pyroptosis (Figure 4F), inhibited the activity of amylase (Figure 4G), reduced release of inflammatory cytokines IL-1β, IL-6, IL-8, and TNF-α (Figure 4H), and decreased the expression of cleaved caspase-1 and caspase-11 (Figure 4I). Our abovementioned results indicate that inhibition of miR-193a-5p significantly enhances caerulein-induced damage (Figures 3B–F), whereas silencing GSDMD reverses the enhancement of caerulein-induced injury by miR-193a-5p inhibitor (Figures 4E–I). These results indicate that miR-193a-5p inhibitor significantly enhances caerulein-induced damage in AR42J cells by increasing GSDMD function.

## Discussion

circRNAs are stable in intracellular and humoral circulation due to resistance to RNase R, providing promising application perspectives as molecular markers or potential therapeutic targets (21). circRNAs also play important roles in many aspects of cell biology, including cell cycle, apoptosis, vascularization, and pyroptosis (22, 23). In this study, we found that the expression of circHIPK3 was significantly elevated in serum of patients with AP and in caerulein-stimulated AR42J cells. circHIPK3 silencing ameliorated caerulein-induced cell damage and inflammatory factor release and inhibited the activation of caspase-1 and caspase-11, which mediate cell pyroptosis.

Studies have shown that circHIPK3 is upregulated in diabetic retinas and retinal endothelial cells (24). Shan K et al. show that circHIPK3 silencing alleviates retinal vascular dysfunction by decreasing inflammation (16). In addition, circHIPK3 silencing alleviated mechanical hyperalgesia and thermal hyperalgesia in STZ-induced diabetes rats by inhibiting neuroinflammation through inhibiting the release of IL-1β, IL-6, IL-12, and TNF-α, indicating a close relationship between circHIPK3 and inflammation (20). Recent findings suggest a key role for non-coding RNAs, especially circRNA and microRNAs (miRNAs), in the progression and management of the inflammatory response through the stability and maintenance of gene expression (25, 26). Several miRNAs, such as miR-124, miR-30a, and miR-193a-5p, have emerged as important transcriptional regulators of some inflammatory mediators (27, 28).

In this study, we determined the levels of miR-124, miR-30a, and miR-193a-5p in blood samples from patients with AP and healthy controls. The level of miR-193a-5p was significantly decreased in patients with AP, while the levels of miR-124 and miR-30a were comparable in patients with AP and healthy controls. In addition, caerulein treatment selectively inhibited the expression of miR-193a-5p, and circHIPK3 knockdown increased miR-193a-5p expression but did not alter levels of miR-124 and miR-30a. Thus, circHIPK3 selectively bound to miR-193a-5p and negatively regulated its expression in acinar cells. Inhibition of miR-193a-5p increased the release of IL-1β, IL-6, IL-8, and TNF-α and activated caspase-1 and caspase-11, thereby counteracting the effects of silencing circHIPK3 on caerulein-induced cell damage. Downregulation of miR-193 has been associated with cancer occurrence (29). The serum miR-193a-5p in epithelium ovarian cancer patients is significantly lower and is correlated with grading and lymph node metastasis (30). Rao et al. found that miR-193 could bind the 3′-UTR of a number of genes that are involved in the regulation of inflammation (31).

In this study, we identified GSDMD as a new target gene of miR-193a-5p. circHIPK3 silencing inhibited and miR-193a-5p inhibitor promoted pyroptosis by regulating GSDMD. Shi et al. demonstrated that activated caspase-1 can cleave GSDMD, which, in turn, initiates cell pyroptosis (32). Pyroptosis is dependent on caspase-1 (33). Caspase-1 specific blockers can inhibit the formation of micropores in the cell membrane of pyroptotic cells, and it is these micropores that lead to a series of morphological changes, such as imbalance of water and electrolyte inside and outside of the cell, cell swelling, cell rupture, and release of inflammatory factors, and then trigger the inflammatory reaction (34). In addition, Man et al. found that caspase-11 directly cleaved Gasdermin D and then mediated pyroptosis, which did not require the involvement of inflammasome (35).

A previous study demonstrated that caspase-1 gene deficiency attenuated the severity of inflammation in AP rats induced by caerulein (36). Injection of caerulein increased the IL-1β mRNA level in wild-type rats, and caspase-1 can convert pro-IL1β into active IL-1β and release it to the outside of the cell (37), recruiting and activating immune cells to induce the synthesis of inflammatory factors and chemokines, thereby enhancing inflammatory responses (38). During AP, acinar cells also release a large number of inflammatory cytokines, such as IL-1β and IL-18, which activate immune cells to induce the infiltration of inflammatory cells, promoting the development of AP, aggravating disease severity, and increasing mortality (39). In this study, we found that caspase-1 and caspase-11 were activated and IL-1β and TNF-α were increased in pancreatic acinar cells after caerulein treatment, suggesting that pyroptosis plays an important role during AP development.

## Conclusion

During AP, upregulation of circHIPK3 inhibits the expression of miR-193a-5p, thereby enhancing GSDMD-mediated pyroptosis in acinar cells, ultimately aggravating AP progression. circHIPK3 may be a useful biomarker for AP diagnosis and a promising therapeutic target for AP treatment.

## Data Availability Statement

All data generated or analyzed during this study are included in this published article.

## Ethics Statement

The studies involving human participants were reviewed and approved by the Clinical Research Ethics Committee of The Third Xiangya Hospital, Central South University. The patients/participants provided their written informed consent to participate in this study.

## Author Contributions

JW, XY, and ZL designed the study. XL, YL, and CP performed the cell biological experiments. HZ and GT performed the reverse transcription-quantitative polymerase chain reaction, western blot, luciferase reporter assay, and immunofluorescence staining assays. All authors contributed to the writing of the manuscript, read, and approved the final manuscript.

### Conflict of Interest

The authors declare that the research was conducted in the absence of any commercial or financial relationships that could be construed as a potential conflict of interest. The reviewer ZL declared a shared affiliation, though no other collaboration, with several of the authors, JW, XL, YL, CP, HZ, GT, XY, ZL, to the handling editor.

## Supplementary Material

Figure S1 The expression of HIPK3 in AR42J cells. qPCR was performed to determine the expression of HIPK3 in AR42J cells after circHIPK3 shRNA transfection.
