# Supplementary figures and images for: CircHIPK3 Promotes Pyroptosis in Acinar Cells Through Regulation of the miR-193a-5p/GSDMD Axis
Source: Front Med (Lausanne). 2020 Apr 7;7:88. doi: 10.3389/fmed.2020.00088 (PMC7154086; doi:10.3389/fmed.2020.00088)

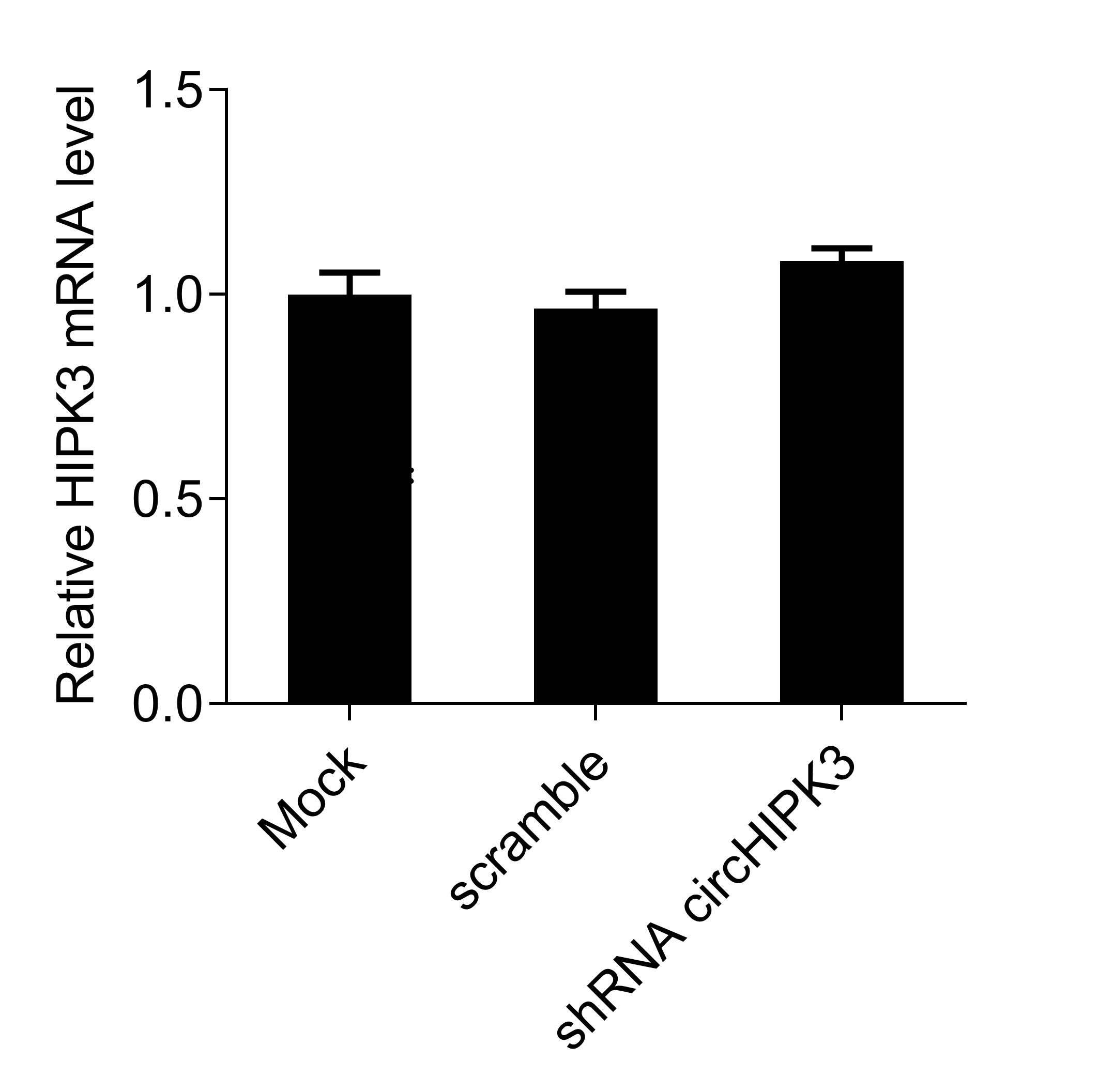

Supplement: Figure S1 — The expression of HIPK3 in AR42J cells. qPCR was performed to determine the expression of HIPK3 in AR42J cells after circHIPK3 shRNA transfection. [file Image_1.TIF]
